# Birt-Hogg-Dubé syndrome encountered at rare lung disease clinic in Anhui province, China

**DOI:** 10.1186/s13023-022-02362-1

**Published:** 2022-05-16

**Authors:** Guofeng Zhang, Jinli Liu, Yushuo Wang, Yue Wang, Xianliang Jiang, Yan Peng, Jun Xiao, Wei Wei, Bing Shen, Long Yi, Jay H. Ryu, Xiaowen Hu

**Affiliations:** 1 Division of Life Sciences and Medicine, Department of Pulmonary and Critical Care Medicine, The First Affiliated Hospital of USTC, University of Science and Technology of China, Hefei, 230001 Anhui China; 2 WanNan Medical College, Wuhu, Anhui China; 3 Division of Life Sciences and Medicine, Department of Dermatology, The First Affiliated Hospital of USTC, University of Science and Technology of China, Hefei, Anhui China; 4 BengBu Medical College, Bengbu, Anhui China; 5 Division of Life Sciences and Medicine, Department of Thoracic Surgery, The First Affiliated Hospital of USTC, University of Science and Technology of China, Hefei, Anhui China; 6 Division of Life Sciences and Medicine, Department of Pathology, The First Affiliated Hospital of USTC, University of Science and Technology of China, Hefei, Anhui China; 7 Division of Life Sciences and Medicine, Department of Urology, The First Affiliated Hospital of USTC, University of Science and Technology of China, Hefei, Anhui China; 8 Division of Life Sciences and Medicine, Department of Radiology, The First Affiliated Hospital of USTC, University of Science and Technology of China, Hefei, Anhui China; 9 School of Basic Medicine, Anhui Medical University, Hefei, Anhui China; 10 Jiangsu Key Laboratory of Molecular Medicine, School of Medicine, Nanjing University, Nanjing, Jiangsu China; 11 Division of Pulmonary and Critical Care Medicine, Mayo Clinic, Rochester, MN USA

**Keywords:** Birt-Hogg-Dubé syndrome, *FLCN* gene, Pneumothorax

## Abstract

**Background:**

Diagnosis of rare diseases remains a challenge in China. We describe our experience with Birt-Hogg-Dubé syndrome (BHDS) encountered at a Rare Lung Disease Clinic recently established in China.

**Methods:**

After the first patient with BHDS was recognized in 2017, a Rare Lung Disease Clinic with a multidisciplinary team of specialists was established. We retrospectively analyzed the data of consecutive patients with BHDS encountered from inception to December 2021.

**Results:**

There were 1, 1, 15, 12 and 21 cases with BHDS diagnosed from year 2017 to 2021, respectively. All 50 patients (34 women) were of Han race with a mean age of 47.4 years. The common manifestations were pulmonary cysts (98%), pneumothorax (54%) and skin lesions (68%). Renal cancer was detected in two patients and renal angiomyolipoma in four other patients. The main presentations leading to diagnosis were pneumothorax (42%), family screening (36%), and lung cysts identified on radiologic imaging (20%). The average delay in diagnosis was 8.3 years, and 4.7 years in patients with only pulmonary cysts. The most frequent pathogenic variant was c.1285del/dup on exon 11 (23%) among 44 patients confirmed by genetic testing. Renal cancer has not been found on follow-up surveillance thus far.

**Conclusions:**

Increasing number of patients with BHDS are being recognized in China, facilitated by establishment of a Rare Lung Disease Clinic. Pulmonary cysts and pneumothorax were commonly encountered features, but skin lesions appeared to be more prevalent in Chinese subjects than previously reported in other Asian countries.

**Supplementary Information:**

The online version contains supplementary material available at 10.1186/s13023-022-02362-1.

## Introduction

Birt-Hogg-Dubé syndrome (BHDS) is a rare autosomal dominant disorder caused by pathogenic variants in the *FLCN* gene located on chromosome 17p11.2 [1]. The disease is characterized by pulmonary cysts often leading to recurrent spontaneous pneumothorax, cutaneous fibrofolliculomas, and an increased risk of renal cell cancers [2]. The earliest families of BHDS were reported by Hornstein and Knickenberg in 1975 [3] and three Canadian physicians in 1977 [4]. In the past 40 years, over 600 families have been reported worldwide.

The prevalence of BHD in the general population has been estimated to be 1.86 cases per million [5]. Therefore, the estimated number of patients with BHDS in the Chinese population (1.4 billion) is 2604. In 2008, the first study of BHDS in China was reported by a research team in Nanjing University, China [6]. By the end of 2021, there were only 221 Chinese patients reported in the literature [7], which is less than 10% of potential BHDS population in China. The average delay from the first symptom to final diagnosis of BHDS has been reported to be 13 years in America and 10 years in China [7, 8]. Misdiagnosis and delayed diagnosis present critical challenges for Chinese patients with BHDS, especially in areas beyond Beijing and Jiangsu Province [7]. Previous reports suggested a lower prevalence of skin lesions among of BHDS patients in Eastern Asian [9, 10]. However, the skin lesions were recently found in almost 50% of confirmed cases from Peking Union Medical College Hospital (PUMCH), a national referral center [11].

A 45-year-old female presenting recurrent pneumothorax and family history of pneumothorax was diagnosed to have BHDS in 2017 at our medical center [12]. To improve the diagnosis and management of patients with rare lung diseases such as BHDS, a Rare Lung Disease Clinic was established by Dr. XH at our hospital in June 2019. This study aims to clarify the characteristics, including the prevalence of skin lesions, in patients with BHDS in Anhui Province, China.

## Methods

We performed a retrospective, descriptive cohort study of patients diagnosed with BHDS at our centre between 2017 and 2021. Based on previous studies on diffuse cystic lung diseases [13–15], suspected patients were initially assessed by an experienced pulmonary physician (Dr. XH) in our Rare Lung Disease Clinic, supported by a multidisciplinary team of specialists including a radiologist (WW), pathologist (YP), dermatologist (JL), urologist (JX) and thoracic surgeon (XJ). When clinical presentation combined with radiological features of pulmonary cysts were suggestive of BHDS, genetic testing was undertaken after patients’ informed consent. The patients who declined participation and who’s diagnosis remained unclear were excluded in this study.

**Fig. 1:**
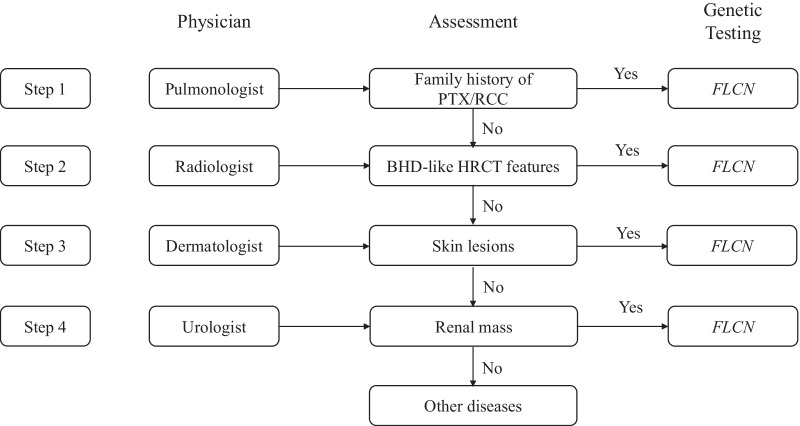
Diagnosis algorithmic approach to BHD syndrome. *PTX* Pneumothorax; *RCC* Renal cell cancer; *HRCT* High-resolution CT scan; BHD-like HRCT features: diffuse cysts with varying shapes located predominantly in the lower lobes and medially (adjacent to the mediastinum)

Our algorithmic approach for the diagnosis of BHDS is depicted in Fig. 1. For patients with personal and/or family history of PTX/RCC, *FLCN* genetic testing was recommended. For patients without personal or family history of PTX/RCC, screening HRCT of the chest was recommended. HRCT features suggestive of BHDS consisted of multiple cysts with varying shapes, located predominantly in the lower lobes and medially. If the diagnosis was still unclear, dermatologic assessment for skin lesions and renal imaging were recommended. When one or more of above steps yielded features suggestive of BHDS, *FLCN* genetic testing was recommended.

The diagnosis of BHDS was based on the criteria proposed by the European BHD consortium. BHDS was diagnosed by fulfilling one major criterion or two minor criteria [16]. The major criteria included: (1) at least five fibrofolliculomas or trichodiscomas (at least one histologically confirmed, of adult onset); (2) pathogenic *FLCN* germline mutation. The minor criteria included: (1) multiple lung cysts (bilateral basally located lung cysts with no other apparent cause, with or without spontaneous pneumothorax); (2) renal cancer (early onset before age 50 or multifocal or bilateral, or mixed chromophobe and oncocytic histology); (3) a first-degree relative with BHDS.

Clinical information including sex, age, family history, pulmonary cysts, detailed history of pneumothorax, skin lesion, renal tumor, genetic testing, diagnosis, treatment, and follow-up was collected.

Chest computed tomography (CT) studies of each patient were assessed by two pulmonary physicians (XH, GZ) independently for the presence of pneumothorax, cysts and cyst localization (upper or lower dominant; central or peripheral dominant), cyst number (< 10, 10–20, > 20), cyst size (maximum diameter: < 1 cm, 1–2 cm, 2–5 cm, > 5 cm), and cyst shape (round to oval, irregular). Renal lesions were evaluated by ultrasound, CT or MRI based on the clinical situation and patient preference. Skin lesions were diagnosed by two physicians specialized in rare respiratory diseases (XH, GZ) and an experienced dermatologist (JL). Skin biopsy was performed when the patient agreed to the procedure. In cases of discrepancies between the three observers, the differences were resolved by discussion and consensus. Family history of pneumothorax was defined as spontaneous pneumothorax in at least 2 family members, including index patients, within three generations. *FLCN* gene analysis was carried out based on methods described in our previous study [17]. Genomic DNA was extracted from peripheral blood leucocytes according to standard procedures and subjected to polymerase chain reaction and Sanger sequencing. The sequencing reactions were conducted on an ABI 3730XL DNA Analyzer. DNA Sequencing Analysis and Sequencing Analysis 5.2.0 software was used to analyze the sequencing results and using Sequencer 5.1 software package for comparative analysis. When a mutation could not be detected by direct sequencing, MLPA analysis and rapid NGS strategy of the *FLCN* gene were used based on our experts’ judgements (Prof. Shen and Dr. Yi) [18].

All data are expressed in the form of means and standard deviations (‾x ± s). SPSS 25.0 was used for data analysis and independent sample t-test was used to compare continuous variables. Fisher’s exact test was used to compare categorical variables. Differences with a value of *P* < 0.05 was considered statistically significant. This single-center and observational study was approved by the Institutional Review Board at the First Affiliated Hospital of University of Science and Technology (Number 2021KY187), and informed consent was obtained from patients.

## Results

One hundred and sixty-six patients with interstitial lung diseases (ILD) and 82 patients with cystic lung diseases were seen in 2021, including 17 BHDS, 14 pulmonary lymphangioleiomyomatosis (LAM), 5 Sjogren’s syndrome and 46 undiagnosed cystic lung diseases, in our Rare Lung Disease Clinic. Fifty patients from 31 families were diagnosed with BHDS at our hospital from January 1, 2017, to December 31, 2021 (Table 1; Additional file 1: Appendix 1). Among 50 patients, sixteen were male and the ratio of male to female was 1:2. The average age at the time of diagnosis was 47.4 ± 11.8 years (range, 18–76 years). Only 3 patients were current smokers. Family screening was available in 11 of 31 identified families and 36% of the 50 patients were diagnosed as a result of family screening.

**Table 1:** Clinical characteristics of study population

Characteristics	(*n* = 50)
Male/female	16/34
Age at examination-yr	47.4 ± 11.8 (18–76)
Pulmonary manifestations	(*n* = 49)
Age at onset of pneumothorax-yr	41.9 ± 10.9 (20–62)
Cysts on chest CT	48/49 (98%)
Pneumothorax	27/50 (54%)
Skin manifestations	(*n* = 47)
Multiple skin-colored papules	32/47 (68%)
FFs and/or TDs confirmed	3/47 (6%)
Renal manifestations	(*n* = 41)
Cancer	2/41 (5%)
AML	4/41 (10%)
Family history of pneumothorax	29/50 (58%)
*FLCN* Genetic testing	(*n* = 45)
Germline mutation	44/45 (98%)

Age was presented as mean ± SD (range)

## Clinical features

**Fig. 2:**
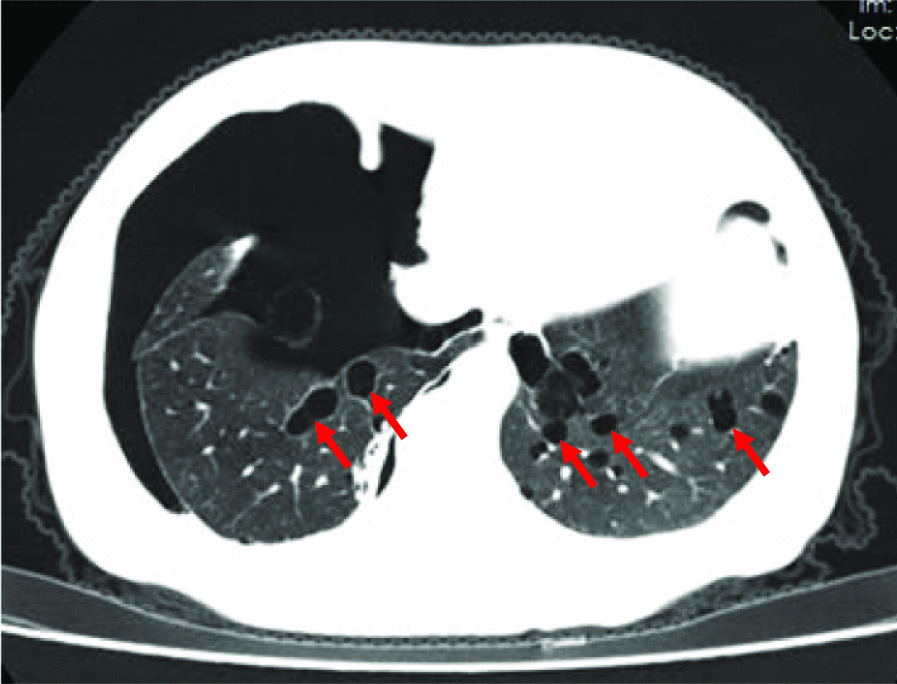
BHD syndrome in a 54-year-old female. Chest CT image shows multiple cysts of varying sizes and irregular shapes predominantly in the lower lungs, and pneumothorax on the right side

Spontaneous pneumothorax had occurred in 27 patients (Fig. 2); the characteristics of pneumothorax were described in 26 cases in detail, including the age at onset of pneumothorax, number of episodes, and side of pneumothorax. Fifty-four symptomatic episodes of pneumothorax had occurred; the mean number of pneumothoraces was 2.1 ± 1.5 (range, 1 to 7). Among these pneumothoraces, there were 3 episodes of bilateral pneumothorax. Family history of pneumothorax was identified in 58% of the families.

Forty-eight patients manifested pulmonary cysts on CT (one patient declined CT imaging) (Table 2). The cystic lesions were located bilaterally in 47 cases (98%) and all manifested lower lung predominance with irregularly shaped cysts. Pulmonary cysts were detected only unilaterally in a thirty-year-old male. In most cases, the maximum diameters of cysts ranged 1–5 cm; the largest cystic lesion measured 8.5 cm. The number of cysts was > 20 in 90% (43/48) of patients and 85% (41/48) presented paramediastinal (medial) cysts. Invasive non-mucinous adenocarcinoma (70% lepidic adenocarcinoma) of the lung (6-1, pT1N0M0, Ia stage) and pulmonary sclerosing pneumocytoma were discovered in two patients, respectively, from the same family. One additional patient was diagnosed with invasive non-mucinous adenocarcinoma of lung (11-1, pT1N0M0, Ia stage) but the detailed typing was unavailable from the outside hospital.

**Table 2:** The characteristics of pulmonary cysts observed on CT

Feature	Cases(*n* = 48)
*Number*	
< 10	2 (4%)
10–20	3 (6%)
> 20	43 (90%)
*Distribution*	
Bilateral to unilateral	
Bilateral	47 (98%)
Unilateral	1 (2%)
Upper to lower	
Upper dominant	0
Lower dominant	48 (100%)
Central to peripheral	
Central dominant	41 (85%)
Peripheral dominant	14 (29%)
Neither central nor peripheral	4 (8%)
Size of maximum cysts	
< 1 cm	1 (2%)
1–2 cm	9 (19%)
2-5 cm	28 (58%)
> 5 cm	10 (21%)
Shape	
Round to oval	2 (4%)
Irregular	46 (96%)

**Fig. 3:**
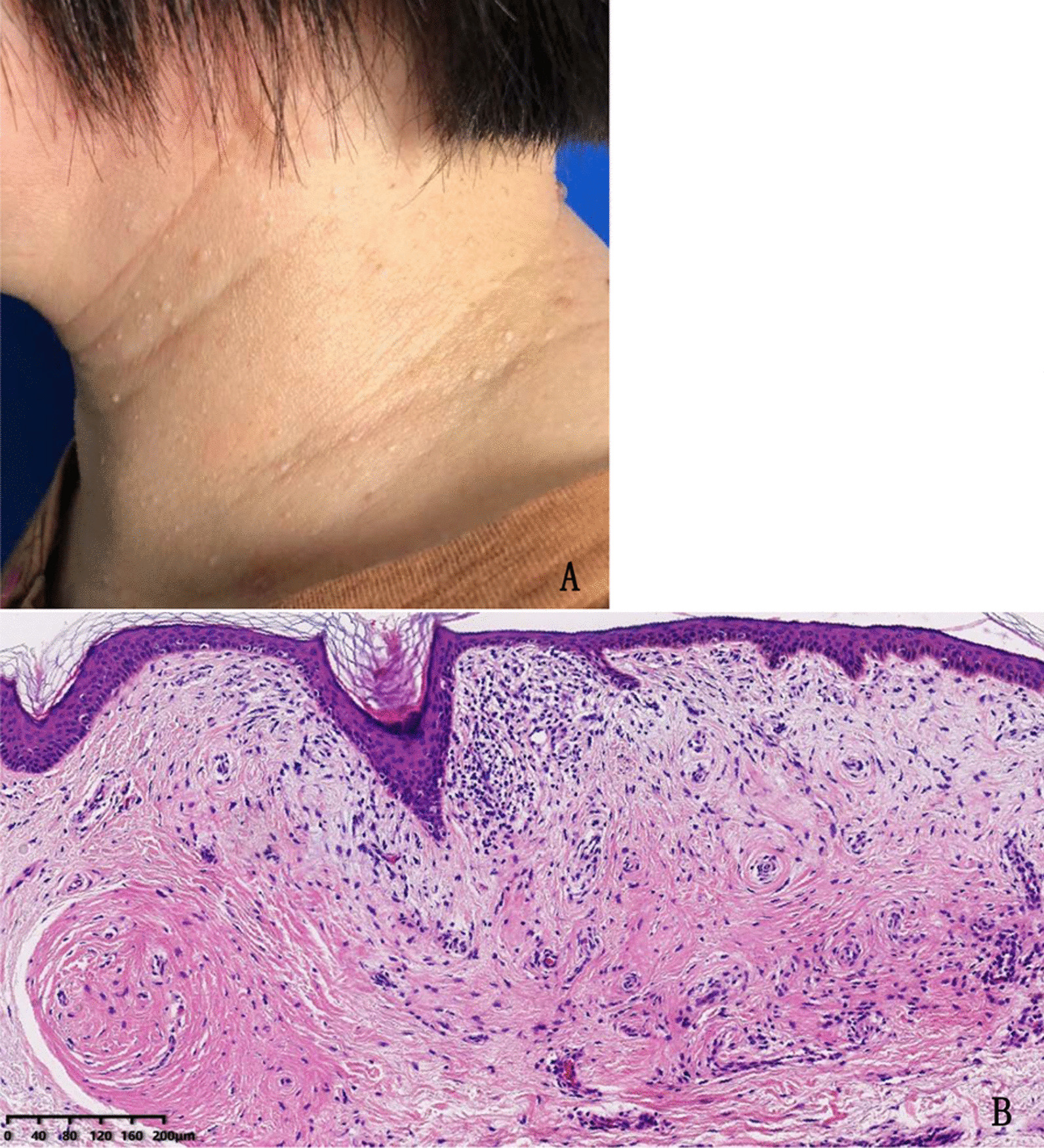
Skin lesions of BHD syndrome observed in a 48-year-old female. **A** Multiple pale and dome-shaped papules on the patient’s neck. **B** Hematoxylin and eosin staining of skin biopsy showed histologic features consistent with trichodiscoma (× 100)

Skin lesions were found in 32/47 (68%) patients and were characterized as skin-colored papules on faces and cervico-thoracic regions (Fig. 3). Nine patients had undergone biopsy which showed fibrofolliculomas in 2, trichodiscoma in 1, and epidermoid cysts in 6 cases. The mean ages were 50.9 ± 9.8 and 37.4 ± 9.6 in patients with skin lesions and those with normal skin, respectively.

Renal tumors were detected in only two (5%) patients. One patient (age 64) underwent a partial nephrectomy which showed a renal chromophobe cell carcinoma. The second patient was diagnosed to have bilateral renal cancer on magnetic resonance imaging (MRI) and is being monitored. In addition, renal angiomyolipoma (AML) was detected in four patients, a type of kidney tumor that is not usually observed in patients with BHDS, and is characteristically associated with tuberous sclerosis complex (TSC). Among these four AML patients, one (6-4) was diagnosed by biopsy, two (8-1 and 12-1) by renal B-ultrasound and the remaining patient (8-2) by contrast-enhanced CT. One of these cases (8-1) subsequently underwent a follow-up MRI which also demonstrated imaging features consistent with AML.

Pathogenic variants in the *FLCN* gene were detected in 44 patients. The most frequent type (23%, 10/44) was the single deletion, duplication of cytosine in codon 1285 of exon 11 (c.1285del, c.1285dup), follow by the mutation of c.1579_1580ins in exon 14 and exon 9 (c.1015C > T) (16%, 7/44). Other mutations occurred much less frequently. Mutation c.1015C > T was associated with a 29% risk (2 of 7 patients) for pneumothorax, and 50% for mutation c.1285dup/del (5 of 10 patients), and 57% for mutation c.1579_1580ins (4 of 7 patients). Mutation c.1015C > T was associated with a 57% risk (4 of 7 patients) for skin lesions, which increased to 71% for mutation c.1579_1580ins (5 of 7 patients), and 90% for mutation c.1285del/dup (9 of 10 patients). However, the differences in the risk of pneumothorax (*P* value, 0.67) and skin lesions (*P* value, 0.35) associated with these three most common mutations were not statistically significant. One patient with renal carcinoma manifested mutation c.1429C > T in exon 12, the other refused genetic testing while her daughter carried mutation c.1381_1382insA. Three novel mutations were detected in three unrelated patients who were all index cases. Pulmonary cysts were detected bilaterally in all these three patients and spontaneous pneumothorax had occurred in one (30-1) patient. Skin lesions were identified in two (27-1 and 30-1) patients and were characterized by skin-colored papules on faces. None of these three patients manifested renal tumors.

## Diagnoses and managements

**Fig. 4:**
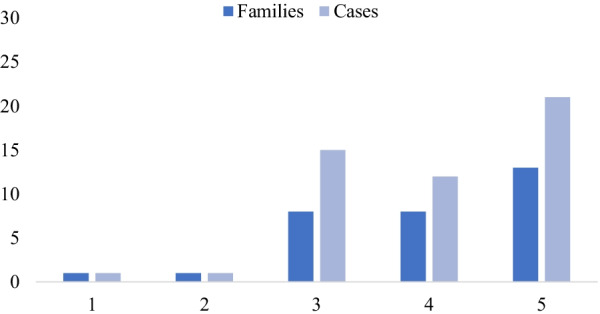
Number of families and cases of BHDS distributed by year of diagnosis

After the first case in 2017, there were 1, 8, 8 and 13 families with BHDS newly diagnosed from 2018 to 2021, respectively (Fig. 4). Except for the first 2 patients, the remainder were diagnosed after the initiation of our Rare Lung Disease Clinic in June 2019. Forty-four patients were confirmed by direct sequencing of the *FLCN* gene and the remaining six by clinical diagnosis. Twenty-one (42%) patients were suspected due to pneumothorax and 18 (36%) cases due to family screening. The remaining 10 (20%) cases were diagnosed by radiologic detection of diffuse pulmonary cysts, except for one patient who manifested a renal mass. Although the age at diagnosis was slightly younger for subjects identified through family screening compared to those identified through index (symptomatic) events, the difference was not statistically significant (44.7 vs. 48.3 years; *P* value, 0.325), likely due to relatively modest number of cases in our cohort. Family screening did result in identification of renal AML in one such (8-2) patient. The prevalence of *FLCN* mutations was 94% (15/16), 43% (15/35), and 54% (14/26) according to presentations of family history of pneumothorax, cystic lung disease, and family screening, respectively. Among of 35 patients presenting with cystic lung disease on HRCT, 5 had pneumothorax.

Almost all initial presentations involved pneumothorax, which was often misdiagnosed as primary spontaneous pneumothorax without the appreciation of the underlying hereditary disorder. The first case of BHDS had been misdiagnosed as LAM. The average interval from initial presentation to eventual diagnosis of BHDS was 8.3 ± 9.1 years (range from 1 to 44 years). The BHDS diagnosis was delayed by 4.7 years for patients presenting with cystic lung disease without a history of pneumothorax. In differentiating BHDS from LAM, serum VEGF-D levels was measured for 17 index BHDS patients and the results ranged from 290 to 640 pg/mL, which are lower than the threshold level for the diagnosis of LAM (≥ 800 pg/mL).

Twenty-four patients with pneumothorax received surgical treatment and pneumothorax recurred in only one case during the follow-up period. Among them, 8 patients with pneumothorax were treated by bullectomy with pleurodesis, 5 by bullectomy alone; the information on the type of surgical procedure was not available in the remaining 11 patients. The recurrence rate of pneumothorax after conservative treatment (including tube thoracostomy) was 22/29 (76%) while the pneumothorax recurred after surgical treatment (bullectomy and/or pleurodesis) in only 1/24 (4%). Partial nephrectomies were performed in one renal cancer patient and another patient refused surgery. All three cases with pulmonary tumors underwent lobectomy.

Fifteen patients underwent annual follow-up visits in our hospital. The average duration of follow-up was 17 months (range, 4–36 months). Spontaneous pneumothorax was detected on chest CT in one patient at her 18-months-follow-up visit and was initially managed conservatively. Six-months later, a large pneumothorax recurred on the same side, and she underwent bullectomy with pleurodesis. No new kidney lesions were detected during the follow-up period.

## Discussion

Striking progress has been achieved with respect to the diagnosis and management of rare diseases in China in recent decades. Two hundred and twenty-two cases with BHDS have been reported up to December 2020 in China [7]; these cases were distributed mostly in the Beijing and Jiangsu Province due to advanced medical resources and better economic conditions present in these regions [7, 19]. To our knowledge, our study describes the largest cohort of patients with BHDS in the Chinese population. The number of patients and families diagnosed in our hospital has increased year by year, especially after the initiation of our Rare Lung Disease Clinic.

Multiple pulmonary cysts detected on chest CT are the most common manifestation of BHDS and have been described in 85–99% of cases [7, 8, 10]. The number, shape, size, and distribution of pulmonary cystic lesions are helpful to distinguish BHDS from other cystic lung diseases [20, 21]. On chest CT, BHDS is characterized by multiple, irregular-shaped cysts of varying sizes with medial and basal predominance in distribution [20, 22, 23]. These features were observed in most of the patients in our study. The detection of lung cysts led to the diagnosis of BHDS in one-fifth of our cases with a relatively shorter delay to eventual diagnosis (4.7 years) compared to those with other presentation modes.

The presence of multiple pulmonary cysts is associated with spontaneous pneumothorax which is 50-times more likely to occur in BHD-affected individuals compared to those not affected, as reported by Zbar and colleagues [24]. In 2008, Ren et al. [6] first demonstrated that *FLCN* mutation contributes to not only familial primary spontaneous pneumothorax but also patients with apparently sporadic (nonfamilial) primary spontaneous pneumothorax. Recent studies suggest that 60–90% of familial primary spontaneous pneumothorax may be attributable to BHDS [19]. Thus, 42% of our patients were suspected due to pneumothorax history. Fifty-four percent of patients had experienced at least one episode of spontaneous pneumothorax and had family history of pneumothorax in our study, which are lower than previous data in China (71% and 85%, respectively) [7]. These differences might reflect the earlier recognition of BHDS in our cohort, prior to onset of pneumothoraces.

In our cohort, BHDS-related pneumothorax was associated with a higher recurrence rate after conservative therapy compared to surgical intervention. Similar findings were observed in both a thoracic surgery cohort reported from Beijing and a patient survey study in America [8, 19]. The high recurrence rate after conservative management may be due to the multiplicity of pulmonary cysts in patients with BHD syndrome, which predispose to recurrent pneumothoraces. Surgical pleurodesis (mechanical abrasion) was recommended in the American Thoracic Society/Japanese Respiratory Society Clinical Practice Guideline to manage pneumothorax in another diffuse cystic lung disease, LAM [25]. Thus, early surgical management should be considered for patients presenting with BHDS-related pneumothorax.

Pulmonary function impairment remains a major concern for patients with cystic lung diseases. Due to constraints on healthcare expenditures, we had only a limited number of follow-ups; no pre-diagnosis PFTs were available. Six BHDS patients performed PFTs and five of them had normal lung function, except one with a slightly reduced Carbon monoxide transfer factor (DLco). Recent study also reported that most patients showed preserved lung function that remained unchanged during follow-up period of up to 6 years [26].

Skin lesions in patients with BHDS usually appear after the age of 20 years, as multiple, dome-shaped, whitish papules on the face, neck, and sometimes trunk [27]. Skin lesions are a common feature observed in Caucasian patients with BHDS, in whom it is found in over 90% [27]. A prior study from China reported a lower prevalence of skin lesions (18%) [7], and similarly observed in other East Asian countries [10, 28]. However, our study shows that skin lesions were present in nearly 70% of patients and suggests this feature may have been overlooked previously (Table 3). This explanation is supported by the observation that the prevalence of skin manifestations in patients with BHDS reported from Peking Union Medical College hospital (PUMCH) increased from 11% in 2017 to 47.2% in 2019 and 54.5% in 2020 [11, 17, 20]. Furthermore, Iwabuchi et al. detected skin lesions in 26/31 (83.9%) Japanese patients with BHDS using dermoscopy and skin biopsy confirmed fibrofolliculoma and/or trichodiscoma histologically in 73.9% [29]. Skin biopsy in the context of an experienced multidisciplinary team is helpful to recognize skin findings associated with BHDS. Thus, recent evidence suggests that the skin involvement in BHDS may not be significantly lower in Chinese patients compared to Caucasians.

**Table 3:** The prevalence of skin lesions in different regions of the world

Author	Years	Region	Cases	The prevalence of skin lesions (%)
Laura S. Schmidt et al. +	2005	Europe	219	84
Nishant Gupta et al.$	2017	America	104	71
Joo Hee Lee et al.&	2019	Korea	12	50
Mitsuko Furuya et al.#	2016	Japan	156	49
Keqiang Liu et al.*	2019	Beijing	39	47
This study	2021	Anhui	47	68

+data from Am. J. Hum. Genet 2005, 76: 1023–1033; $data from Ann Am Thorac Soc 2017, 14(5): 706–713; &data from Korean J Intern Med 2019, 34(4): 830–840; #data from Clin Genet 2016, 90: 403–412; *data from Orphanet J Rare Dis 2019, 15; 14(1): 223

Renal cancer is the most serious complication of BHDS, are often bilateral and multifocal, with the oncocytoma and chromophobe variant being the most common types of malignancy [30]. The risk of developing renal tumors is seven-fold higher in patients with BHDS, compared to the general population [24]. The low prevalence of renal involvement in our cohort may be due to selection bias since nearly all patients in our study were recruited from the respiratory department. Another reason might be relative lack of long-term follow-up in our cohort. However, further studies on potential racial differences int the prevalence of renal cancer may be warranted. For example, Elke C. Sattler et al. recently reported BHDS to be associated with early-onset colorectal cancer in their German cohort compared to controls (5.1% vs. 1.5%, *P*-value 0.0068) [31]. However, another study reported no increased prevalence of colorectal carcinoma in patients with BHDS [32]. In view of conflicting data and medical spending restraints, we did not routinely perform colon cancer screening. We intend to explore the association of colorectal cancer with BHDS in future studies.

The most frequent mutations associated with BHDS in our study were c.1285del/dup on exon 11, similar to the results in previous studies [33, 34]. Toro et al. reported that 48% mutations were identified in exon 11, as a mutation ‘hotspot’ for BHDS [34]. The most frequent mutation in Chinese BHDS patients was the single deletion, duplication of cytosine in codon 1285 of exon 11 as reported from PUMCH [11]. Mutation c.1015C > T accounted for about 1/6 of patients in this study and three novel mutations of c.1381_1382insA in exon 12, c.761 T > C in exon 7, c.1283_1284insA in exon 11 were encountered. Mutation was not detected in a forty-three-year-old female who manifested fibrofolliculomas by skin biopsy. Toro et al. reported that patients with mutation in exon 9 or 12 have a higher risk of pneumothorax compared to those carrying mutation in other exons [34]. A recent study from Germany reported that the mutation c.250-2A > G was associated with twice as high risk of pneumothorax compared with the mutation c.1285dup [33]. However, the correlation between genotype and phenotype was not found in our study. This issue needs to be explored further with larger number of patients.

It was reported that 399 patients with BHDS were identified in Netherlands with a population of about 17.1 million [32]. Anhui Province, located in Eastern of China with 61-million citizens, would be estimated to have over 1400 patients with BHDS. There has been no previous data regarding the actual prevalence of BHDS in China. Although our study cohort in Anhui Province comprises the largest number of cases of BHDS described from China, it only accounts for 3.6% of estimated BHDS cases in our Province. This highlights the under-recognition of rare diseases in developing regions of the world. Establishment of a Rare Lung Disease Clinic supported by a multidisciplinary team at our medical center led to improved recognition and diagnosis of BHDS over a recent 2.5-year period. Similar model of care may allow better care of patients with rare diseases in other developing regions of the world.

There were several limitations to our study. Firstly, the number of cases is modest in this cohort. However, considering the rarity of disease we have collected a relatively large number of cases in a single center. Secondly, due to the retrospective nature of this study, the family history/screening and annual follow-up evaluations were not always available. Thirdly, skin biopsies were performed on only 9 patients as many individuals refused to undergo this procedure. However, all the skin lesions were examined by a same multidisciplinary team focused on diffuse cystic lung diseases. A long-term, muti-center prospective study is needed to allow better understanding of this rare disease that will improve management.

## Conclusion

There has been increasing number of patients with BHDS recognized in China, facilitated by establishment of a Rare Lung Disease Clinic. Pulmonary cysts and pneumothorax were commonly encountered features, but skin lesions appeared to be more prevalent in Chinese subjects than previously reported in other Asian countries.

## Supplementary Information

**Additional file1: Appendix 1** Clinical characterizations and mutation analysis of BHD syndrome patients in Anhui province, China.

## Data Availability

The datasets generated and analyzed for this study are not publicly available due to participant privacy but are available from the corresponding author upon reasonable request.
