# Correction to: Birt-Hogg-Dubé syndrome encountered at rare lung disease clinic in Anhui province, China

**DOI:** 10.1186/s13023-022-02438-y

**Published:** 2022-08-11

**Authors:** Guofeng Zhang, Jinli Liu, Yushuo Wang, Yue Wang, Xianliang Jiang, Yan Peng, Jun Xiao, Wei Wei, Bing Shen, Long Yi, Jay H. Ryu, Xiaowen Hu

**Affiliations:** 1 Department of Pulmonary and Critical Care Medicine, The First Affiliated Hospital of USTC, Division of Life Sciences and Medicine, University of Science and Technology of China, Hefei, Anhui 230001 China; 2 WanNan Medical College, Wuhu, Anhui China; 3 Division of Life Sciences and Medicine, Department of Dermatology, The First Affiliated Hospital of USTC, University of Science and Technology of China, Hefei, Anhui China; 4 BengBu Medical College, Bengbu, Anhui China; 5 Division of Life Sciences and Medicine, Department of Thoracic Surgery, The First Affiliated Hospital of USTC, University of Science and Technology of China, Hefei, Anhui China; 6 Division of Life Sciences and Medicine, Department of Pathology, The First Affiliated Hospital of USTC, University of Science and Technology of China, Hefei, Anhui China; 7 Division of Life Sciences and Medicine, Department of Urology, The First Affiliated Hospital of USTC, University of Science and Technology of China, Hefei, Anhui China; 8 Division of Life Sciences and Medicine, Department of Radiology, The First Affiliated Hospital of USTC, University of Science and Technology of China, Hefei, Anhui China; 9 School of Basic Medicine, Anhui Medical University, Hefei, Anhui China; 10 Jiangsu Key Laboratory of Molecular Medicine, School of Medicine, Nanjing University, Nanjing, Jiangsu China; 11 Division of Pulmonary and Critical Care Medicine, Mayo Clinic, Rochester, MN USA

## Correction to: Orphanet Journal of Rare Diseases (2022) 17:203 https://doi.org/10.1186/s13023-022-02362-1

The authors of the original article [1] would like to clarify that the official name and address for affiliation 1 is the following:

Department of Pulmonary and Critical Care Medicine, The First Affiliated Hospital of USTC, Division of Life Sciences and Medicine, University of Science and Technology of China, Hefei, Anhui, 230001, China.

This is reflected in the 'Author details' section of this Correction. The original article has been updated accordingly.
