# Supplementary material for: Birt-Hogg-Dubé syndrome encountered at rare lung disease clinic in Anhui province, China
Source: Orphanet J Rare Dis. 2022 May 16;17:203. doi: 10.1186/s13023-022-02362-1 (PMC9112470; doi:10.1186/s13023-022-02362-1)
Supplement: Supplementary file 1 — Additional file1: Appendix 1 Clinical characterizations and mutation analysis of BHD syndrome patients in Anhui province, China. [file 13023_2022_2362_MOESM1_ESM.docx]

| Appendix 1: Clinical characterizations and mutation analysis of BHD syndrome patients in Anhui province, China | | | | | | | | | | | | |
| --- | --- | --- | --- | --- | --- | --- | --- | --- | --- | --- | --- | --- |
| Patient/family No. | Sex | Age | Smoking | Family history | Spontaneous pneumothorax | Lung cysts | Skin lesions | Renal lesions | Mutation (exon) | Mutation (cDNA level) | Mutation (protein level) | Follow-up  (month) |
| 1 | F | 41 | N | Y | Y | Y | N | N | 10 | c.1165G>T | p. Glu389* | N |
| 2 | F | 47 | N | N | N | Y | N | N | 4 | c.208G>T | NA | 36M, St |
| 3-1 | F | 49 | N | N | N | Y | Y | N | 9 | c.1015C>T | p. Gln339Ter | 28M, St |
| 3-2 | F | 45 | N | N | N | Y | N | NA |  |  |  | N |
| 3-3 | M | NA | N | N | Y | Y | NA | NA | NA |  |  | N |
| 4 | F | 32 | N | N | Y | Y | N | NA | 6 | c.469_471  delTTC | p. Phe157del | N |
| 5-1 | M | 65 | N | Y | Y | Y | Y | N | 9 | c.1015C>T | p. Gln339Ter | 18 |
| 5-2 | M | 30 | N | Y | N | Y | N | N |  |  |  | N |
| 6-1 | F | 63 | N | Y | Y | Y | Y | N | 14 | c.1579_1580insA | p. Arg527Glnfs*75 | 35M, recurrent Left pneumothorax 2 times |
| 6-2 | F | 48 | N | Y | N | Y | Y | N |  |  |  | 17M, St |
| 6-3 | F | 56 | N | Y | N | Y | Y | N |  |  |  | N |
| 6-4 | F | 54 | N | Y | Y | Y | Y | AML |  |  |  | 17M, St |
| 6-5 | F | 47 | N | Y | Y | Y | Y | NA |  |  |  | N |
| 6-6 | M | 40 | N | Y | N | Y | N | N |  |  |  | N |
| 6-7 | F | 24 | N | Y | Y | Y | N | N |  |  |  | N |
| 7 | F | 32 | N | N | N | Y | N | N | 14 | c.1597_1598  delCA | p. Gln533Glufs*68 | N |
| 8-1 | M | 45 | Y | N | N | Y | Y | AML | 10 | c.1177-5_1177-3delCTC | - | N |
| 8-2 | F | 54 | N | N | N | Y | Y | AML |  |  |  | 17M, St |
| 9 | M | 64 | Y | Y | Y | Y | Y | N | 11 | c.1285dupC | p. His429Profs*27 | N |
| 10 | F | 42 | N | Y | Y | Y | N | NA | 10 | c.1177-5_1177-3delCTC | - | N |
| 11-1 | F | 48 | N | Y | Y | Y | Y | N | 11 | c.1285delC | p. His429Thrfs*39 | N |
| 11-2 | M | NA | N | Y | N | NA | N | N |  |  |  | N |
| 12-1 | F | 44 | N | Y | Y | Y | Y | AML | 7 | c.761T>C# | p. Leu254Pro | 14M, St |
| 12-2 | M | 76 | N | Y | Y | Y | Y | N | NA |  |  | 15M, St |
| 13 | M | 40 | N | N | Y | Y | Y | N | 1,2,3 | Exon1,2,3 | - | N |
| 14-1 | M | 49 | N | Y | Y | Y | N | N | 1,2,3 | Exon1,2,3 | - | N |
| 14-2 | F | 58 | N | Y | Y | Y | Y | N | NA |  |  | 23M, St |
| 14-3 | F | 47 | N | Y | N | Y | Y | N | NA |  |  | N |
| 15 | F | 50 | N | N | Y | Y | NA | NA | 1,2,3 | Exon1,2,3 | - | N |
| 16 | F | 29 | N | Y | Y | Y | N | NA | 10 | c.1165G>T | p. Glu389Ter | N |
| 17 | F | 56 | N | N | N | Y | Y | N | 11 | c.1285dupC | p. His429Profs*27 | 9M, St |
| 18 | F | 50 | N | N | Y | Y | N | NA | 7 | c.761T>C# | P. Leu254Pro | 7M, St |
| 19 | F | 49 | N | Y | N | Y | Y | N | 11 | c.1285dupC | p. His429Profs*27 | N |
| 20-1 | F | 54 | N | Y | Y | Y | Y | N | 11 | c.1285delC | p. His429Thrfs*39 | N |
| 20-2 | M | 37 | N | Y | N | Y | Y | N |  |  |  | N |
| 21-1 | F | 45 | N | N | N | Y | N | N | 12 | c.1381_1382  insA# | p. Ser461Lysfs*28 | 7M, St |
| 21-2 | F | 64 | N | N | N | Y | Y | right renal chromophobe cell carcinoma | NA |  |  | 4M, St |
| 22 | F | 43 | N | N | Y | Y | Y | N | ND |  |  | 4M, St |
| 23-1 | F | 43 | N | Y | Y | Y | Y | N | 9 | c.1015C>T | p. Gln339Ter | N |
| 23-2 | M | 18 | N | Y | N | N | N | N |  |  |  | N |
| 24 | F | 48 | N | N | Y | Y | Y | N | 9 | c.946_947delAG | p. Ser316Tyrfs*73 | N |
| 25 | F | 54 | N | Y | N | Y | Y | NA | 9 | c.1015C>T | p. Gln339Ter | N |
| 26 | F | 24 | N | N | Y | Y | Y | N | 7 | c.634C>T | p. Gln212* | N |
| 27 | M | 53 | Y | Y | N | Y | Y | N | 7 | c.761T>C# | p. Leu254Pro | N |
| 28 | M | 56 | N | Y | Y | Y | Y | N | 11 | c.1285delC | p. His429Thrfs*39 | N |
| 29-1 | M | 70 | N | N | N | Y | NA | bilateral renal cancer | 12 | c.1429C>T | p. Arg477* | N |
| 29-2 | F | 45 | N | N | N | Y | Y | N |  |  |  | N |
| 30 | F | 51 | N | Y | Y | Y | Y | N | 11 | c.1283_1284  insA# | p. Pro428Hisfs*20 | N |
| 31-1 | F | 57 |  | N | Y | Y | Y | N | 11 | c.1285dupC | p. His429Profs*27 | N |
| 31-2 | M | 36 |  | N | N | Y | Y | N |  |  |  | N |

#Novel mutation; NA not available; ND not detected; AML renal angiomyolipoma; St stable
